# Lean Path for High-Quality Development of Chinese Logistics Enterprises Based on Entropy and Gray Models

**DOI:** 10.3390/e21070641

**Published:** 2019-06-28

**Authors:** Yimin Huang, Qiuxiang Li, Xueying Wang, Hongna Wang

**Affiliations:** 1 School of Management and Economics, North China University of Water Resources and Electric Power, Zhengzhou 450046, China; 2 Institute of Management Science and Engineering, Henan University, Kaifeng 475004, China; 3 Business School, Henan University, Kaifeng 475004, China

**Keywords:** high-quality development, management innovation, entropy weight-TOPSIS, gray system, lean

## Abstract

According to literature review and the data of China’s logistics listed companies, this paper firstly designs the high-quality development evaluation system of logistics enterprises and establishes the panel data model group. Secondly, the method of entropy weight-Technique for Order Preference by Similarity to an Ideal Solution (TOPSIS method) is used to synthesize and regress the indexes, and obtains that the fitting degree of the model is low, which is caused by the lack of data of some indicators in the logistics enterprises. Due to the gray nature of data information, the improved gray relational model and the three-dimensional gray relational model are constructed to study, in-depth, the strategic focus and breakthrough of high-quality development of Chinese logistics enterprises. The research finds that the innovation and the operation ability of Chinese logistics enterprises are weak, which shows specifically in the following aspects: (1) The irrational structure of the employees, the proportion of employees with a bachelor degree or above is small, and the high-education personnel fail to significantly promote the corporate performance; (2) R&D expenditure has little effect on the high-quality development of enterprises. The proportion of R&D expenses is small and cannot be translated into actual benefits, and the ability of enterprise management innovation is insufficient. According to these findings, this paper gives three lean paths for the high-quality development of China’s logistics enterprises.

## 1. Introduction

**Figure 1:**
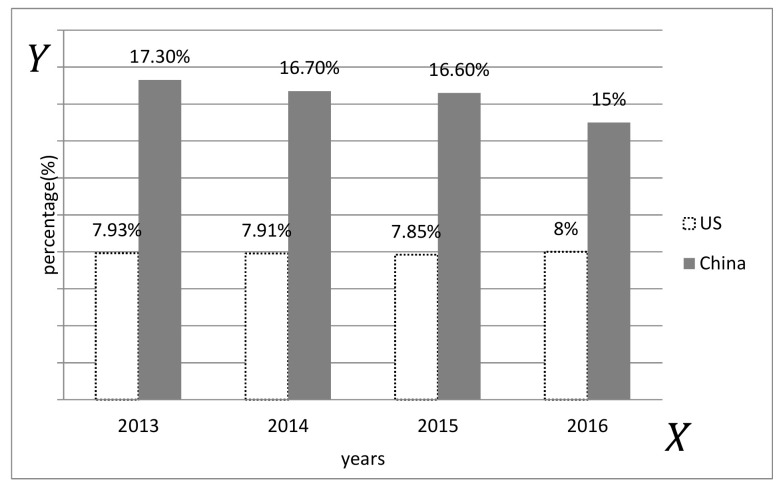
Sino-US logistics costs as a percentage of GDP. (According to the research report 2017).

The nineteenth session of national congress of the communist party of China has given a new strategic orientation to the development of logistics in the new era, integrating logistics into the scope of infrastructure development and entering the strategic and basic areas of national priority development. The implementation of the “One Belt and One Road” strategy, the new urbanization, the upgrading of the manufacturing industry, the reform of the railway and highway system, and other key points of the national economic development and reform strategy are inseparable from the strong support of the logistics industry [1]. However, the following problems still exist in the development of China’s logistics industry: (1) High cost (see Figure 1) and low efficiency. China’s social logistics costs accounted for a gradual decline in the proportion of GDP, and the logistics industry has made great progress, but the cost proportion is still higher than the developed countries [2]. The low logistics efficiency is the main reason for the high logistics cost [3]; (2) low marketization. At present, China’s own enterprise logistics system still occupies a dominant position in the entire logistics market. A series of logistics activities, from raw material procurement to finished product sales, mainly rely on the internal organization of the production enterprise to complete itself; however, the third-party logistics has a low degree of marketization; (3) insufficient service innovation. In order to achieve good logistics services, enterprises often choose a number of logistics companies to cooperate, which indicates that the most of the logistics companies only have a single service method, and few companies provide comprehensive and integrated logistics services. It can only provide basic logistics services such as transportation and warehousing, but has not yet fully developed high-level logistics services such as logistics information services, logistics system planning and design, and supply chain consulting. Logistics enterprises are mostly in a traditionally-simple and low-threshold competitive environment. The characteristics of logistics enterprises indicate that the development of China’s logistics industry has not yet entered the “high-quality fast lane”; however, the high-quality development of the logistics industry is imperative.

However, the high-quality development of the logistics industry is inseparable from the high-quality development of logistics enterprises. Then, it is urgent to theoretically solve what are the foundations and conditions for the high-quality development of logistics enterprises, and where the breakthroughs in the high-quality development of logistics enterprises are, which are the problems and starting points that need to be solved in this paper. Through literature review, the paper constructs a high-quality evaluation system for logistics enterprises, and uses the econometric model and gray spatial relevance model to study the high-quality development of Chinese logistics listed companies. This paper aims to reveal the key factors that restrict the high-quality development of Chinese logistics enterprises, and to design a high-quality development path for logistics enterprises based on lean theory, which provides theoretical support for the high-quality development of logistics enterprises.

## 2. Design of High-Quality Development Evaluation System for Logistics Enterprises

The objective of this paper is to promote the high-quality development of logistics enterprises, improve the level of the overall logistics industry, and lay a good foundation for China’s economic development. It is necessary to establish a scientific evaluation index system for logistics enterprises, guide enterprises to make decisions, operate, and manage in a scientific way. Further this paper explores the key factors that restrict high-quality development of Chinese logistics enterprises by means of mathematical modeling, and find a breakthrough in the high-quality development of Chinese logistics enterprises. At present, many scholars have carried out research on the enterprise evaluation system. Zhao and Zeng [4] built a corporate evaluation index system based on the innovation level, including seven aspects: innovation investment, research and development, innovative production, and enterprise operational capability, which is a more practical three-level evaluation indicator system. On the basis of in-depth study of existing theories and methods, Wang et al. [5], based on the balanced scorecard theory, established an enterprise performance evaluation index system suitable for the background of informationization. The above literature show that China’s current research on enterprise evaluation index system is quite extensive, and the index system is different in terms of its establishment. However, the enterprise competitiveness evaluation index system proposed by them does not involve high-quality development concepts.

### 2.1. Connotation of High-Quality Development

Guided by the new concept of innovation and coordinated development, high-quality development takes promoting people’s well-being as its starting point and foothold, and focuses on solving the problem of unbalanced development in China. With the concept of “quality first, efficiency first,” people should also pay attention to speeding and keeping the economy running within a reasonable range. With the supply-side structural reform as the main line, people will reconstruct China’s supply system at the four levels of industry, products, enterprises, and factors to achieve matching with demand. At the same time, high-quality development is a development method which promotes total factor productivity by means of innovation-driven production. Ren believed that high-quality development was an advanced and optimal state of economic development quality [6], not simply pursuing economic aggregates and economic growth, but paying more attention to balanced development of economy, society, and environment, achieving higher quality and more efficient and more sustainable development [7]. Li [8] pointed out that high-quality development meant high-quality supply, high-quality demand, high-quality configuration, high-quality input and output, high-quality income distribution and high-quality economic cycle. The realization of high-quality development should be guided by a new development concept, which means that innovation is the first driving force, coordination is the endogenous feature, green is the universal form, openness is the only way, and sharing is the fundamental goal. Feng [9] found that the promotion of China’s high economic speed to high-quality development requires the government, enterprises, and society to perform their duties and cooperate to form a social synergy. For the government, the most important thing is to tighten up the market economy system, to turn economy to high-quality development practical system foundation. Jia [10] put forward the “four musts” to achieve high-quality development including: Must grasp the strategic goal of building a modern economic system; must implement the principle of quality first and efficiency first; must base on supply-side structural reform; and efforts must be made to build a market mechanism to form an economic system with a dynamic micro-subject and a macro-control.

### 2.2. Design of High-Quality Development Evaluation System for Logistics Enterprises

Scholars have studied the high-quality development of the logistics industry and have obtained rich research results. Su et al. [11] constructed the green development strategy model and performance evaluation index system of delivery enterprises expression. Le [12] pointed out five transformation and upgrading ideas and five development strategies for cold chain logistics in the supply-side structural reform background. Shi [13] believed that in order to achieve sustainable development, logistics enterprises should strengthen the standardization of logistics facilities, promote modern logistics technology, raise government service awareness, reduce institutional costs and innovate logistics personnel training mode. Xie and Shuai [14] believed that the key task of high-quality development was to increase effective supply to lead demand, eliminate the inefficient supply to adjust the structure, to reduce costs and to improve efficiency, and pointed out that logistics enterprises of different types and scales should be based on their own characteristics and resources. This paper designs a high-quality development evaluation system for logistics enterprises (see Table 1 for details).

(1) Corporate social recognition indicators

1) Enterprise value can measure the ability of all shareholders to create and manage value, and is the external performance of the listed company’s intrinsic value [15], which reflects the market’s perception and development expectations of the company during a certain period, and this paper uses the market value of enterprises to total assets. 2) Corporate social contribution rate includes three agents: government, employees, and investors. Under the background of high-quality development, logistics enterprises should not only pay attention to the economic benefits of enterprises, but also pay attention to social responsibilities, correctly handling and coordinating the interests between the company and the government, internal and external interest groups, which is an important factor to realize the sustainable development of the enterprises. 3) Brand perception is moving towards a high-quality development stage, promoting quality change, and promoting “the transformation of Chinese products to Chinese brands”. Therefore, enterprises should strive to promote brand building. This paper uses the ratio of intangible assets of enterprises to total assets.

(2) Enterprise scale efficiency indicators

1) Total assets which is reflecting the company’s size, ability of resisting risks, and profiting. 2) Main business cost ratio. The high-quality of enterprises cannot be measured by the scale of assets alone, the focus should be on the efficiency of enterprises, so that the whole enterprise can obtain higher output on the basis of less investment. The higher the index, the lower the profit contribution rate of the main business income, and the low efficiency of capital input and output. 3) The per capita profit rate reflects the profit generated by each employee of the enterprise, and measures the relationship between human resources investment and profit output.

(3) Enterprise innovation performance indicators

Promoting the high-quality development of enterprises requires the driving force for enterprise development be transformed from one-single-factor input to more innovation-driven factors. 1) The number of patents measures the knowledge innovation ability of enterprises to innovate in knowledge. 2) The intensity of market development investment is to consider the ability of enterprises using the intellectual property rights to develop markets and generate income in industry. This article measures the proportion of sales expenses to total operating income. 3) Intangible assets growth. The rate is used to measure the level of innovation management of enterprises and the sustainability of innovation capabilities.

(4) Enterprise innovation investment indicators

1) The proportion of R&D personnel reflects the investment of innovative human resources. 2) The proportion of R&D expenses reflects the innovation and financial input of enterprises. 3) The structure of employees reflects the content and structure of intellectual capital held by enterprises, which is also an important factor affecting enterprise innovation, having high-quality employees can improve the company’s innovation performance [16,17].

(5) Enterprise operational capability indicators

High-quality development requires enterprises to improve operational capabilities and to enhance operational efficiency. 1) Fixed asset turnover rate; 2) net assets return rate, measuring the efficiency of the company’s use of free capital, reflecting the ability of obtaining net income of its own capital to obtain net income; 3) assets and liabilities are closely related to the operation ability [18]. The better the operation ability is, the less the pressure the company’s capital chain is; and the better the credit is, the easier to get creditors’ attention and trust.

## 3. Measurement Model and Analysis

Many domestic and foreign scholars have studied the relationship between enterprise performance and its influencing factors through the econometric model, for example, Beal and Yasai-Ardekani [39] constructed an econometric model and used regression analysis to test the matching of the CEO’s individual functional experience with the corporate competitive strategy. Jia et al. [40] used SPSS software to analyze the impact of the matching of executive competency and strategic orientation on corporate performance. Wang et al. [41] took the 2009–2014 Chinese listed companies as a research sample and empirically tested the impact of lean inventory management on firm performance by constructing the panel data model. The results show that there is an inverted U-shaped relationship between lean inventory management and firm performance. Therefore, the econometric model is used for empirical research in this paper to find the key factors that restrict the performance development of logistics enterprises.

### 3.1. Theoretical Mechanism and Research Hypothesis

#### 3.1.1. Relationship between Indicator X and Indicator Y

The index X belongs to the affecting corporate performance indicators and the index Y is the Measuring corporate performance indicators. Many scholars have studied the relationship between the two. For example, Schumpeter [42] indicated that the enterprise R&D expenditure or R&D expenditure announcement could have a positive impact on the market value of the enterprise. Meanwhile, some scholars proposed that if the technological innovation of the enterprise was insufficient or unsustainable and the R&D capability is not formed, the investment would become a sunk cost and reduce the value of the enterprise [43]. For the enterprise efficiency indicators, most scholars proved that there was a positive correlation between enterprise innovation investment and enterprise efficiency, but some scholars believed that technological innovation could not improve enterprise efficiency [44,45,46]. This is because some R&D investments do not take into account potential customer needs, so innovation investment had not been converted into corporate benefits [47,48]. Regarding the indicators of corporate social responsibility, Liu pointed out that when the return on net assets of enterprises was high, enterprises might abandon the narrow concept of pursuing their own interests and turn to the fulfillment of corporate social responsibility [20], so there was a significant positive correlation between corporate social contribution rates and the net profit margin of enterprises. Ding pointed out that the company’s net profit growth rate and the company’s fixed asset turnover rate were important factors affecting its operating efficiency (the ratio of main business costs) [27]. Pan [33] proposed that the market development intensity of Chinese pharmaceutical companies could reflect the innovation investment of enterprises. Tou [49] believed that the total asset turnover rate of enterprises had a positive correlation with corporate performance. The above literature have discussed the relationship between index X and Y. This paper considers the establishment of the model group of index X and index Y in the context of logistics enterprises, and puts forward the following assumptions:

H1: The corporate performance indicators $X_{ij},\;\left(i=1,\;2\;j=1,\;2,\;3\right)$ have a significant positive impact on the measurement of corporate performance indicators $Y_{mn}$ $\left(m,n=1,2,3\right)$.

#### 3.1.2. The Relationship among Indicators Y

Corporate brand perception is determined by the ratio of intangible assets to total assets. Many domestic and foreign researches showed that the intangible assets of enterprises are significantly and positively correlated with the value of enterprises. Xu and Wang [50] conducted an empirical study with Chinese listed companies as a sample, and concluded that the intangible assets disclosed by listed companies had a positive impact on stock prices. Through empirical research Moskowitz concluded that the value of a company depends on its social contribution rate through empirical research. The greater the corporate social contribution is, the greater its corporate value is [51]. Ruf et al. [52] found that there was a positive correlation between corporate social responsibility and corporate value. Another point of view was that enterprises’ fulfillment of social responsibilities would reduce the value of enterprises, which is because enterprises undertook social responsibilities, and enterprises’ costs would inevitably increase, leading to a decline in corporate profits and corporate value [53]. Chen [54] pointed out that the social responsibility and value correlation between different industries were very different and could not be generalized. Based on the analysis above, in order to explore the impact of corporate brand perception and social contribution rate on corporate value in the logistics industry, this paper proposes the following assumptions:

H2: Corporate brand perception $Y_{11}$ and corporate social contribution rate $Y_{12}$ both have significant positive impact on corporate value $Y_{13}$

### 3.2. Selection of Data, Variables, and Model Selection

In this paper, 114 enterprises of A-share logistics listed companies in Shanghai and Shenzhen stock exchanges from 2013 to 2017 were chosen as initial research enterprises, giving a total of 572 samples. When sorting out the data, it was found that due to the late start of the logistics industry, the disclosed information of many companies was incomplete, and the data of R&D personnel and R&D expenditure were seriously missing. After filling the data by using scientific and reasonable methods, the abnormal values were eliminated. Finally, 174 valid samples were obtained for research. These data were mainly obtained from Wind Financial Information Software, Juchao information network, and the state intellectual property office. These data were used to construct an econometric model to empirically analyze hypothesis proposed above. The main variables and calculation method involved in the study can be referred to in the indicator system in Table 1. At the same time, when studying the relationship among indicators Y, joining the two control variables of firm size and corporate capital structure were considered. The size of the enterprise was measured by the total assets of the enterprise. The capital structure of enterprises, measured by the company’s asset-liability ratio, found that enterprises with high market value generally have higher anti-risk ability, and their capital structure ratio tends to be higher.

The measurement model set in this article is as follows:

(1) $$\mathrm{Model}\;\text{1–9}:\;Y_{ij}=\beta_{0}+\beta_{1}SS+\beta_{2}RDP+\beta_{3}RDE+\beta_{4}FAT+\beta_{5}ROE+\beta_{6}ALR+\mu_{1},$$

(2) $$\mathrm{Model}\;10:\;EV=\alpha_{0}+\alpha_{1}SCR+\alpha_{2}BP+\alpha_{3}TA+\alpha_{4}ALR+\mu_{2}$$

where $X_{ij}=BP,SCR,EV,TA,OCR,PCP,NOP,MD,IAGR$. $\beta_{0},\;\alpha {\;}_{0}$ are constant terms in the model, which represent the parts that are not interpreted by the independent variables and exist for a long time (non-random), that is, information residual. In addition, $\mu_{1},\;\mu_{2}$ are random error terms in the model, which are the errors of the predicted value and the actual value of the constant term removed in the independent variable interpretation space. $\beta_{1}$ to $\beta_{6}$, $\alpha_{1}$ to $\alpha_{4}$ are a number of unknown parameters, reflecting the degree of influence of each independent variable on the dependent variable.

### 3.3. Test Results and Analysis

In this paper, the analysis method of panel data was used. Due to the availability of data, the selected time interval was not very long, and forty-five logistics listed companies in the period 2013–2017 were selected to form a “short and wide” unbalanced panel. Gao [55] believes that the “short and wide” panel data is more special and could not be tested by unit root. Besides, in order to eliminate the data dimension, the data were normalized in this paper.

Using Eviews 9.0 software, this paper directly operated the panel data as follows: Pearson correlation coefficient was used to analyze the correlation of each variable to reflect the degree of linear correlation between two variables. If the degree of correlation between the independent variables was high, it indicated the existence of multiple collinearity. The mixed model and the fixed-effect model were first compared by F test, and the fixed-effect model and the random effect model were compared by Hausman test. Then the selected model was finally determined. The results are shown in Table 2.

The research found that: (1) In model 1–9, except for model 3 and 9, the fitness of these models is higher than 0.7, which indicates that the indicator X selected in the context of logistics enterprises can explain indicator Y better, so the indicator X can be seen as an important factor affecting the high-quality development of enterprises. (2) There is a significantly negative correlation between the proportion of R&D expenses of enterprises and the per capita profit of enterprises. It indicates that the increase of innovation investment will reduce the value and efficiency of enterprises, whereas consistent with the research results of Chen et al. [43]. This is because the innovation investment can be regarded as the cost expenditure of the enterprise, while the innovation investment of the logistics enterprise does not achieve effective transformation, which may be the lack of enterprise management innovation ability. (3) There is a significantly negative correlation between corporate social contribution rate and corporate value shown in model 10, which also verifies the conclusion of Vance et al. that the degree of corporate social responsibility in China is relatively low, which is regarded as a cost expenditure and fails to enhance the value of enterprises [49]. There is a significant positive correlation between brand perception and corporate value. Intangible assets can become the leverage of tangible assets value-added, improve production efficiency, save resources, and accelerate and magnify the role of tangible assets. Intangible assets can be directly converted into productivity, enhance enterprise value, by means of technological innovation, brand strategy, and other methods.

## 4. Gray Spatial Association Model and Analysis

According to the analysis, it can be found that there is a high degree of correlation between the indicators $Y_{ij}$, but most scholars only analyzed the influencing factors of a specific indicator among the indicators $Y_{ij}$. Although it was easy to understand, the results obtained are too one-sided. This paper considered using the entropy weighted-Technique for Order Preference by Similarity to an Ideal Solution (TOPSIS method) to synthesize the indicators to evaluate enterprise performance into a single index, expecting to evaluate logistics enterprises more comprehensively.

The entropy method is a method to determine the weight of each index according to the amount of information provided by the indicator data. Entropy is a term in thermodynamics. In information theory, entropy is a measure of the degree of disorder of the system. Generally speaking, in the evaluation, if the numerical variation degree of the index is larger, the smaller the entropy value is, the more information is included and transmitted, and the corresponding weight is larger; otherwise, the weight of the index is smaller, so the indicators can be utilized. The entropy value is used to calculate the weight, which is the entropy weight.

The TOPSIS method is a commonly-used multi-objective decision analysis method. Its main principle is to sort according to the closeness of the evaluated object and the idealized solution, and to evaluate the relative merits of the existing objects. As a multivariate statistical method for evaluating problems, compared with the traditional evaluation method, the TOPSIS method has the advantages of intuitive principle, simple calculation. In this paper, the entropy weight method and the TOPSIS method were combined to establish a comprehensive evaluation index, which effectively solves the shortcomings that the weights of each sub-index are difficult to determine and leads to the low accuracy of the evaluation results. It effectively evaluates the high-quality development of logistics enterprises, is easy to operate, and the result is highly accurate.

Assuming that n indexes of m sample logistics enterprises are evaluated as $x_{ij}$, where i is the sample logistics enterprise, and j is the jth index, establish the initial matrix A:

(3) $$A=\left(\begin{array}{ccc} x_{11} & \cdots & x_{1n}\\ \vdots & \ddots & \vdots \\ x_{m1} & \cdots & x_{mn} \end{array}\right)$$

(1) Normalize the matrix A

(4) $$\left\{\begin{array}{cc} y_{ij}=\frac{x_{ij}-minx_{ij}}{maxx_{ij}-minx_{ij}} & i\in \left[1,m\right]\;\;\;j\in I_{1}\\ y_{ij}=\frac{maxx_{ij}-x_{ij}}{maxx_{ij}-minx_{ij}} & i\in \left[1,m\right]\;\;\;j\in I_{2} \end{array}\right.$$

There $I_{1}$ is the profitability index, the bigger the value; $I_{2}$ is the cost index, the smaller the value.

(2) Calculate the entropy weight of each index by information entropy

Determine the weight of each index, and set the entropy of the jth index as $H_{j}$, then

(5) $$H_{j}=-k\sum_{i=1}^{m}{\begin{array}{c} p_{i,j}lnp \end{array}}_{i,j},\;\;\;\;j\in \left[1,m\right]$$

(6) $$p_{ij}=\frac{y_{ij}}{\sum_{i=1}^{m}y_{ij}}\;\;k=\frac{1}{lnm}$$

Plus, when $p_{ij}=0$, ${\begin{array}{c} p_{i,j}lnp \end{array}}_{i,j}=0$.

According to entropy, the entropy weight of the j th index is solved, that is

(7) $$\omega_{j}=\frac{1-H_{j}}{n-\sum_{j=1}^{n}H_{j}},\;\;\;\;0\leq \omega_{j}\leq 1,\;\;\;\;\sum_{j=1}^{n}\omega_{j}=1.$$

(3) Find the normalized matrix Z. The normalized matrix Y is multiplied by the index weight ω to obtain the weighted normalized matrix Z.

(8) $$Z=\left(\begin{array}{ccc} z_{11} & \cdots & z_{1n}\\ \vdots & \ddots & \vdots \\ z_{m1} & \cdots & z_{mn} \end{array}\right)=\left(\begin{array}{ccc} \omega_{1}y_{11} & \cdots & \omega_{n}y_{1n}\\ \vdots & \ddots & \vdots \\ \omega_{1}y_{m1} & \cdots & \omega_{n}y_{mn} \end{array}\right)$$

(4) Calculate the positive and negative ideal solutions 

Given that each index is forward processed, the positive ideal value $z_{j}^{+}$ and negative ideal value $z_{j}^{-}$ of each index correspond to the maximum value and minimum value of the corresponding index, respectively, namely $z_{j}^{+}=max\left(z_{1j},z_{2j},\ldots ,z_{nj}\right)$ and $z_{j}^{-}=min\left(z_{1j},z_{2j},\ldots ,z_{nj}\right)$.

(5) Calculate the distance from each sample to positive ideal solution and negative ideal solution, $D_{i}^{+},\;D_{i}^{-}$, namely $D_{i}^{+}=\sqrt{\sum_{j=1}^{n}{\left(z_{ij}-z_{j}^{+}\right)}^{2}}\;i\in \left[1,m\right]$ and $D_{i}^{-}=\sqrt{\sum_{j=1}^{n}{\left(z_{ij}-z_{j}^{-}\right)}^{2}}\;i\in \left[1,m\right]$.

(6) Calculate the comprehensive single index $C_{i}$ $\left(C_{i}=\frac{D_{i}^{-}}{D_{i}^{-}+D_{i}^{+}}\right)$. The larger the index is, the better the performance is.

The synthesized indexes were regressed and the results are shown in Table 3:

However, after using the econometric model for the regression of the composite single indexes, it was found that the fitting degree was generally low, and the regression coefficient was not significant. Considering the problems of data, because there is a serious lack of the indexes of R&D personnel and R&D cost in logistics enterprises, the ratio method may have a great impact on the composite index after the ratio method is adopted in this paper. At the same time, the method of mathematical model was used to study economic problems. Therefore, the gray space correlation model was introduced to study the composite index of 46 samples with complete data.

### 4.1. Model Construction

#### 4.1.1. Model 11 (Gray Superiority Factor Analysis Model)

The gray superiority factor analysis model was used to find out and classify the superiority factors affecting the high-quality development of China’s logistics industry. The calculation steps are as follows:

**Definition** **1.**  *Assume that* $X_{i}=(X_{i}\left(1\right),X_{i}\left(2\right),L,X_{i}\left(n\right)$ *are sequences of data representing a system’s characteristics,* D *is a sequence operator,* $XD=\left(x\left(1\right)d,x\left(2\right)d,l,x\left(n\right)d\right)$ *, where* $x_{i}\left(k\right)d=\frac{x_{i}\left(k\right)}{x_{i}\left(1\right)},x_{i}\left(1\right)\neq 0,k=1,2,L,n$ *, then* XD *is called the image after the calculation of initialing operator D, which is also called initial image for short.*

**Definition** **2.**  *Assume that *$x_{i}=\left(x_{i}\left(1\right),x_{i}\left(2\right),L,x_{i}\left(n\right)\right)$*are sequences of data representing a system’s characteristics, set*$X_{i}^{0}\left(k\right)=x_{i}\left(k\right)-x_{i}\left(1\right),k=1,2,L,n$*, then*$x_{i}^{0}=\left(x_{i}^{0}\left(1\right),x_{i}^{0}\left(2\right),L,x_{i}^{0}\left(n\right)\right)$*is called initial image of*$X_{i}$.

**Definition** **3.**  *Assume that*

(9) $$\left\{\begin{array}{c} s_{i}=\int_{1}^{n}\left(X_{i}-x_{i}\left(1\right)\right)dt\\ s_{j}=\int_{1}^{n}\left(X_{j}-x_{j}\left(1\right)\right)dt\\ s_{i}-s_{j}=\int_{1}^{n}\left(X_{i}^{0}-X_{j}^{0}\right)dt \end{array}\right.$$

*and* $\pi_{ij}=\frac{1+\left|S_{i}\right|+\left|S_{j}\right|}{1+\left|S_{i}\right|+\left|S_{j}\right|+\left|S_{i}-S_{j}\right|}$ *,* $\pi_{ij}$ *measures the absolute relations of sequences.*

**Definition** **4.**  *Assume that sequences* $X_{i},X_{j}$ *, are in the same length and the initial values are not zero, and,* $X_{i}^{\prime },\;X_{j}^{\prime }$ *, are initial images of* $X_{i},\;X_{j}$ *, respectively, then,* $\lambda_{ij}=\frac{1+s_{i}^{\prime }+s_{j}^{\prime }}{1+s_{i}^{\prime }s_{j}^{\prime }+{\left|s_{i}^{\prime }-s_{j}^{\prime }\right|}^{\prime }}\;\lambda_{ij}$ *are used to measure the sequences relations of the change rates relative to the initial point.*

**Definition** **5.**  *When there are more than one relative sequence and main sequence, respectively, in gray relational analysis, superior analysis must be used to identify which are superior factors and which are not. If there are*n*parent sequences which are denoted as:*$\left\{X_{1}\right\},\left\{X_{2}\right\},L,\left\{X_{m}\right\}$*, and*m*subsequences which are denoted as*$\left\{Y_{1}\right\},\left\{Y_{2}\right\},L,\left\{Y_{n}\right\}$*. Following Definition 3 and 4, the correlation matrixes of the parent sequences for each sub sequence, respectively, are called *$R_{j},\;R_{x}$:

(10) $$R_{j}=\left[\begin{array}{c} \begin{array}{cccc} \pi_{11} & \pi_{12} & L & \pi_{1m}\\ \pi_{21} & \pi_{22} & L & \pi_{2m}\\ M & M & M & M\\ \pi_{n1} & \pi_{n2} & L & \pi_{nm} \end{array} \end{array}\right],\;R_{x}=\left[\begin{array}{cccc} \lambda_{11} & \lambda_{12} & L & \lambda_{1m}\\ \lambda_{21} & \lambda_{22} & L & \lambda_{2m}\\ M & M & M & M\\ \lambda_{11} & \lambda_{11} & L & \lambda_{nm} \end{array}\right]$$

**Definition** **6.**  *Determining the effects of subsequences on parent sequences based on the relational coefficients of* $R_{j},\;R_{x}$ *, factors with significant influence are called superior factors, the corresponding parent sequence or subsequences are called superior parent sequences or superior subsequences.*

#### 4.1.2. Model 12 (Three-Dimensional Gray Correlation Model)

**Definition** **7.**  *Let* $x_{i}$ *be the system factor, its value at the point* $\left(i,j\right)$ *in space is* $a_{ij}$ *, there* i≤M,j≤N,M,N *are constants,* $A_{i}={\left(a_{ij}\right)}_{M\times N}$ *is the system behavior matrix.*

**Definition** **8.**  *Let the system behavior matrix be *$A_{i}={\left(a_{ij}\right)}_{M\times N}$*, and the behavior surface corresponding to*${\left(a_{ij}\right)}_{M\times N}=\left[\begin{array}{cc} a_{11} & \begin{array}{cc} \ldots & a_{1N} \end{array}\\ \begin{array}{c} \vdots \\ a_{M1} \end{array} & \begin{array}{cc} \begin{array}{c} \ddots \\ \ldots \end{array} & \begin{array}{c} \vdots \\ a_{MN} \end{array} \end{array} \end{array}\right]$*be*$X=\left\{Ax+By+C|x\in \left[i,i+1\right],y\in \left[j,j+1\right],i=1,2,\cdots M-1,;j=1,2,\cdots M-1\right\}$.

**Definition** **9.**  *Let the system behavior matrix be* $A={\left(a_{ij}\right)}_{M\times N}$ *, D be the matrix operator, and* $AD={\left(a_{ij}d\right)}_{M\times N}$ *; in which* $a_{ij}d=a_{ij}-a_{i1}$ *. The matrix operator D is the beginning edge zeroing operator whose unification behavior matrix is* $A={\left(a_{ij}\right)}_{M\times N}$ *, and AD is the beginning edge zeroing image whose unification behavior matrix is* $A={\left(a_{ij}\right)}_{M\times N}$ *, denoted as:* $A_{i}^{0}=XD={\left(a^{0}{}_{ij}\right)}_{M\times N}$ *.*

**Definition** **10.**  *Set the system behavior matrix for* $A_{i}=a_{ij}{}_{M\times N}$ *zero beginning edge like for* $A_{i}^{0}=XD=a^{0}{{}_{ij}}_{M\times N},$ *corresponding to initial image of the surface for X ^ 0, s* $=\int_{1}^{M}\int_{1}^{N}X^{0}dxdy.$

**Definition** **11.**  *Set the system behavior matrix to *$A_{i}={\left(a_{ij}\right)}_{M\times N}$*, D is a matrix operator,*$AD={\left(a_{ij}d\right)}_{M\times N}$*; inside*aijd=aijai1*. The matrix operator D is called the initial value operator of the unified behavior matrix as*$={\left(a_{ij}\right)}_{M\times N}$*, and AD is the initial value image of the unified behavior matrix as*$A={\left(a_{ij}\right)}_{M\times N}$*, denoted as:*$A_{i}^{c}=XD={\left(a^{c}{}_{ij}\right)}_{M\times N}$.

**Definition** **12.**  *Let us set two behavior matrices *$A_{p}={\left(a_{ij}\right)}_{M\times N}$*,*$A_{q}={\left(a_{ij}\right)}_{M\times N}$*, their initial zero images are *$A_{p}^{0}={\left(a^{0}{}_{ij}\right)}_{M\times N}$*,*$A_{q}^{0}={\left(b^{0}{}_{ij}\right)}_{M\times N}$*; then*$s_{p}-s_{q}=\int_{1}^{M}\int_{1}^{N}\left(A_{p}^{0}-A_{q}^{0}\right)dxdy$.

**Definition** **13.**  *Let us set two behavior matrices* $A_{p}={\left(a_{ij}\right)}_{M\times N}$ *,* $A_{q}={\left(a_{ij}\right)}_{M\times N}$ *is a homogeneous matrix, their initial zero images are* $A_{p}^{0}={\left(a^{0}{}_{ij}\right)}_{M\times N}$ *,* $A_{q}^{0}={\left(b^{0}{}_{ij}\right)}_{M\times N}$ *; then the absolute correlation between the two matrices* $A_{p}={\left(a_{ij}\right)}_{M\times N}\;and\;A_{q}={\left(a_{ij}\right)}_{M\times N}$ *is:*

(11) $$\varepsilon_{pq}=\frac{1+\left|s_{p}\right|+\left|s_{q}\right|}{1+\left|s_{p}\right|+\left|s_{q}\right|+\left|s_{p}-s_{q}\right|}.$$

**Definition** **14.**  *Let us set two behavior matrices *$A_{p}={\left(a_{ij}\right)}_{M\times N}$*,*$A_{q}={\left(a_{ij}\right)}_{M\times N}$*is a homogeneous matrix, the corresponding initial values are*$A_{p}^{c}$*and*$A_{q}^{c}$*, respectively. The relative correlation between matrix*$A_{p}={\left(a_{ij}\right)}_{M\times N}$*,*$A_{q}={\left(a_{ij}\right)}_{M\times N}$ is

(12) $$\varepsilon_{pq}^{c}=\frac{1+\left|s_{p}^{c}\right|+\left|s_{q}^{c}\right|}{1+\left|s_{p}^{c}\right|+\left|s_{q}^{c}\left|+\right|s_{p}^{c}-s_{q}^{c}\right|}$$

**Definition** **15.**  *Let us set two behavior matrices *$A_{p}={\left(a_{ij}\right)}_{M\times N}$*,*$A_{q}={\left(a_{ij}\right)}_{M\times N}$*is a homogeneous matrix, their initial zero images are*$A_{p}^{0}={\left(a^{0}{}_{ij}\right)}_{M\times N}$*,*$A_{q}^{0}={\left(b^{0}{}_{ij}\right)}_{M\times N}$*; then the absolute correlation between the two matrices*$A_{p}={\left(a_{ij}\right)}_{M\times N}\;and\;A_{q}={\left(a_{ij}\right)}_{M\times N}$ is

(13) $$\varepsilon_{pq}^{0}=\frac{1+\left|s_{p}^{0}\right|+\left|s_{q}^{0}\right|}{1+\left|s_{p}^{0}\right|+\left|s_{q}^{0}\left|+\right|s_{p}^{0}-s_{q}^{0}\right|}$$

### 4.2. Empirical Analysis

According to the index system, setting series of $Y_{11\;},Y_{12},\;Y_{13},\;Y_{21},\;Y_{22},\;Y_{23},\;Y_{31},\;Y_{32},\;Y_{33}$ as system characteristic behavior sequences; setting series of $X_{11\;},\;X_{12},\;X_{13},\;X_{21},\;X_{22},\;X_{23}$ as correlation factor behavior sequences; $Y_{1\;},\;Y_{2},\;Y_{3}$ as system characteristic behavior matrixes, and $X_{1\;},\;X_{2}$ as correlation factor behavior matrixes. The $M_{ij}$ matrix from model 12 is:

$$M_{ij}=\left(\begin{array}{c} \begin{array}{cc} .5040 & .6185\\ .3035 & .3788\\ .6217 & .5078 \end{array} \end{array}\right)$$

Using the advantage analysis model, we get the following forum from the $M_{ij}$ matrix:

$\gamma_{11}=0.5040>\gamma_{12}=0.6185$,
$\gamma_{21}=0.3035>\gamma_{22}=0.3788$,
$\gamma_{32}=0.6217>\gamma_{31}=0.5078$,
$\sum_{i=1}^{3}\lambda_{i2}=1.5051>\sum_{i=1}^{3}\lambda_{i1}=1.4292$.

The following conclusions can be obtained:

(1) The effect of enterprise operation ability on the growth of enterprise social recognition is significantly higher than that of enterprise innovation input.
(2) The effect of enterprise operation capability on the growth of enterprise scale efficiency is significantly higher than that of enterprise innovation input, but the correlation between the two indexes and enterprise scale efficiency is less than 0.5. It shows that the two indicators do not contribute much to the growth of the enterprise scale efficiency, and the enterprise operation ability and innovation input need to be strengthened.
(3) The effect of enterprise innovation input on the growth of enterprise innovation performance is significantly higher than that of enterprise operation ability.
(4) The whole capacity of corporate social contribution rate is greater than the value of innovation investment, which is in conformity with the truth of the logistics enterprises in our country; China’s logistics enterprises started late, and most of them are single-function enterprises. The phenomenon of product homogeneity is serious. They often take customers through price war among enterprises. This objectively requires enterprises to improve their logistics operation ability, reduce operating costs and expand profit margins.

Therefore, the lack of attention to innovation has become a key factor restricting the high-quality development of enterprises. In order to explore which specific indicators in the innovation input and operation ability of enterprises restrict the high-quality development of enterprises, the matrix $R_{ij}$ is introduced in this paper. The matrix $R_{ij}$ is obtained from model 11

$$R_{ij}=(\begin{array}{cc} \begin{array}{ccc} .5865 & .9711 & .6554\\ .5152 & .5933 & .5273\\ .6174 & .8471 & .7109 \end{array} & \begin{array}{ccc} .7694 & .7379 & .6277\\ .5474 & .5418 & .8441\\ .8657 & .8229 & .5941 \end{array}\\ \begin{array}{ccc} .5985 & .9139 & .6769\\ .5267 & .6635 & .5479\\ .9038 & .9038 & .6183 \end{array} & \begin{array}{ccc} .8067 & .7708 & .6122\\ .583 & .5733 & .9146\\ .705 & .681 & .6679 \end{array}\\ \begin{array}{ccc} .5854 & .9769 & .6535\\ .5545 & .8342 & .5979\\ .7533 & .6609 & .9551 \end{array} & .\begin{array}{ccc} .7661 & .735 & .6293\\ 6697 & .6498 & .7028\\ .8169 & .8589 & .5436 \end{array} \end{array})$$

From the matrix we can see that:

$$\sum_{i=1}^{9}\lambda_{i2}=7.3647>\sum_{i=1}^{9}\lambda_{i4}=6.5299>\sum_{i=1}^{9}\lambda_{i5}=6.3714>\sum_{i=1}^{9}\lambda_{i6}=6.1363>\;\sum_{i=1}^{9}\lambda_{i3}=5.9432>\sum_{i=1}^{9}\lambda_{i1}=5.6413.$$

Hence the following conclusions can be obtained:

(1) The indicators affecting the overall social value of Chinese logistics enterprises can be divided into three gradients. The first gradient includes the proportion of R&D personnel and the turnover rate of fixed assets. The second gradient is the return on equity and asset-liability ratio. The third gradient is R&D expenditure and staff structure. The influence gradually decreases with the increase of gradient.
(2) The proportion of R&D personnel and the turnover rate of fixed assets have the greatest impact on the overall social value of enterprises. R&D staff is the foundation of enterprise product innovation, the greater the proportion of R&D personnel is, the stronger R&D capability is, the faster R&D SPEED, and the higher the success rate is, so the enterprise R&D personnel proportion can produce great influence on the enterprise overall performance [1]. The fixed asset turnover mainly react on the enterprise equipment utilization degree, whereby the higher the utilization is, the more cost is reduced, thus bringing obviously higher benefit. These two indicators are closely related to the overall social value of the enterprise. It is of great significance to continue increasing the proportion of researchers and improving the turnover rate of fixed assets to achieve high-quality development of the enterprise.
(3) The return on equity and asset-liability ratio have the second largest impact on the overall social value of logistics enterprises. The return on equity mainly reflects the profitability of enterprises, which means that profitability has a certain impact on the overall social value of logistics enterprises, but it needs to be further improved. The asset-liability ratio mainly reflects the long-term solvency of the enterprise, representing the capital structure of the enterprise, and is closely related to its operational capacity, and the enterprise needs to further optimize it.
(4) The R&D expense and the staff structure weakly influence the logistics enterprise overall social value influence. However, high-quality development requires enterprises to take innovation as the first driving force, to strengthen research and to development expenditure, and optimize enterprise staff structure, so these two indicators need to be improved.

## 5. Lean Path Design for High-Quality Development of Chinese Logistics Enterprises

### 5.1. Analysis of the Key Factors Restricting the High-Quality Development of Chinese Logistics Enterprises

Through the above analysis, it was found that the four key factors restricting the development of China’s logistics industry are: serious shortage of logistics professionals, insufficient investment in innovation, weak innovation transformation ability, and low overall operational efficiency and benefit.

(1) The most critical factor restricting the high-quality development of China’s logistics enterprises is the structure of employees. It shows that the structure of employees in logistics enterprises is seriously unreasonable. The main reasons are as follows:
  1) The number of employees with a bachelor degree or above is small. According to the collected data, the average indicator is only 23.32. A survey of logistics occupation types conducted by Orleans State University shows that about 92% of US logistics managers have a bachelor’s degree, while 41% have a master’s degree, and 22% have a formally professional qualification certificate. It can be seen that China is far behind the foreign level.
  2) The establishing of logistics majors in China’s universities is relatively late, and there are problems such as the mismatch between college training objectives and corporate demand skills. This has led to a shortage of logistics management personnel, logistics planning personnel, and logistics research personnel who really understand management and have modern logistics management concepts. However, under the background of high-quality development, logistics companies are required to use innovation as the driving force and optimize the talent structure of enterprises. Therefore, enterprises should pay great attention to them.
(2) Another key factor restricting the high-quality development of enterprises is the proportion of R&D expenses. R&D expenses have little impact on the overall social value of enterprises, and cannot effectively drive the high-quality development of logistics enterprises. The existing logistics enterprises data shows that the companies pay insufficient attention to R&D expenses, and the proportion of R&D expenses to the main business income is generally less than 1.5%.
(3) The results of logistics enterprises show that the R&D expenses of logistics enterprises have not been transformed into enterprise innovation ability, thus the enterprise innovation investment has not been effectively utilized. The reason is that the enterprise management innovation ability is insufficient.
(4) Analysis shows that the contribution rate of enterprise operation ability to the overall social value of the enterprise is better than that of the innovation input, but the driving force for the high-quality development of the enterprise is still weak, and the operational capability is mainly measured by the financial indicator profit, indicating that the current low efficiency in operation leads to poor efficiency in Chinese logistics enterprises, which is consistent with the survey results of the project team on logistics enterprises.

### 5.2. The High-Quality Development Lean Path of Chinese Logistics Enterprises

**Figure 2:**
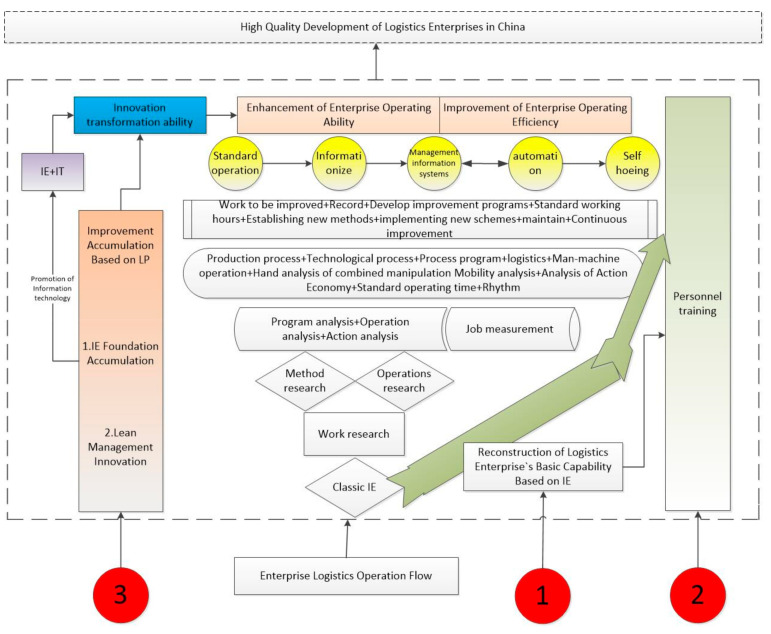
Lean path for high-quality development of China’s logistics industry.

A lean path suitable for the high-quality development of Chinese logistics enterprises is put forward based on the enterprise research and survey, through the enterprise research and visits. This paper designed a lean path suitable for the high-quality development of Chinese logistics enterprises, as shown in Figure 2. The combination of IE (industrial engineering) theory and Toyota’s production practices has creatively evolved the TPS (Toyota production system), later known as “lean production” (LP), and has become a powerful force driving almost every industry change in the world. The use of LP to reconstruct the basic capabilities of logistics enterprises is a continuous improvement process that requires constant accumulation [56]. Figure 2 shows the “inverted pyramid” path that supports the continuous improvement of China’s logistics industry. In the “inverted pyramid” path of logistics enterprise basic capability reconstruction, IE (LP) is the foundation.

By means of the IE theory, the company conducts operation research and method research on the enterprise logistics production process, deeply analyzes the basic laws that the logistics production process should follow, comprehensively improves the process, reduces waste, and forms standard operations. The company introduces modern information technology and builds an efficient management information system through “IE+IT,” where IT is information technology, and continuously improves the efficiency of the company. At the same time, in the continuous cycle of the above process, it can attach importance to human factor, strengthen the training of logistics professionals, and make up for the shortage of professional talents. Through the process of continuous operation, it can improve the company’s operational capabilities and achieve high-quality development. Based on this, this paper designed three paths for the high-quality development of Chinese logistics companies.

Path one: Reorganizing path of basic ability for high-quality development of logistics enterprises in China based on industrial engineering. Through the construction of model and panel data test, this paper found that the overall basic ability of Chinese logistics enterprises is weak, so advanced information technology cannot be carried out otherwise the effect is not good. This is mainly due to the existence of many problems in the production process of logistics enterprises exposed by in-depth exploration and corporate visits, such as waiting, inventory, overproduction, and other waste. Without good processes there will be no good standard operations, which leads to basic capabilities of enterprises being weaker and being unable to form a channel for the release of productivity brought about by modern technology, and sometimes even negatively affect enterprises. China’s logistics enterprises, as well as China’s manufacturing industry and its service industries, have similar problems. Therefore, under the requirements of high-quality development, people must pay attention to the reconstruction of basic capabilities and open the way for the implantation of advanced technologies.

Path two: A talent development path based on a continuous improvement process. According to the research results, it was found that the key factor restricting the development of China’s logistics industry is the problem of logistics professionals, which is consistent with the conclusions of the company’s research. From the perspective of the development process of Chinese logistics enterprises, enterprises started late, and fewer professionals were trained in practice. At the same time, the quality of talents trained in college logistics majors is not high, because students trained in efficient logistics do not have the skills required by society. These undergraduate and above professionals also need the training of enterprise practice to become the high-level talents needed for the high-quality development of real logistics enterprises. Therefore, companies must have such a platform to enable logistics professionals or other employees with a bachelor degree or above to grow on this platform. This platform is a continuous improvement platform for enterprise infrastructure reconfiguration. The employees can only continue to fight against waiting inventory overprices and other waste to improve the business process, in order to grow into the professional talents needed by the company. The cultivation of talents must go through the entire business improvement process, as shown in Figure 2.

Path three: The path of improving the ability of enterprise innovation transformation based on the management innovation method. Innovation is a major obstacle to the high-quality development of Chinese logistics companies. Enterprises do not have good basic capabilities, and without excellent professionals with practical training, there will be no good ability to innovate and transform. No matter how much investment such an enterprise pays to innovation, it will not produce good results. Therefore, it is imperative to improve the ability of enterprises to innovate and transform, which also is something that businesses need to keep an eye on. The improvement of innovation and transformation ability depends on the improvement of the basic ability of the enterprise, and the improvement of the basic ability is the result of the integrated application of the management innovation methods, as shown in Figure 2.

The three paths are designed to eliminate all kinds of unnecessary waste in the logistics operation process, improve the efficiency of input and output, and realize the process of lean logistics. The three paths together form a system for upgrading soft power in the context of high-quality development of enterprises.

## 6. Conclusions

In order to find a scientific path suitable for the high-quality development of China’s logistics industry, the key factors that restrict the high-quality development of China’s logistics industry must be found out firstly. After the key factors are analyzed by the econometric model, it is found that there is a strong correlation between the performance evaluation indicators of enterprises. Although using a single indicator to evaluate the performance of enterprises is simple and easy to understand, the scientific nature is biased (the key factors of the high-quality development of China’s logistics industry are not independent, but synergistic. This is a breakthrough in the design and evaluation methods of the evaluation system, the research findings are in line with the reality.) Therefore, the method of entropy weight-TOPSIS is used to synthesize and regress the indexes, which shows that the fitting degree of the model is low. Therefore, the improved gray relational model and three-dimensional spatial gray relational model are constructed, and the strategic key points and breakthroughs of the high-quality development of China’s logistics enterprises are studied in depth. The research finds that (1) the structure of enterprise employees is unreasonable, the proportion of employees with a bachelor degree or above is small, and highly educated personnel fail to significantly promote enterprise performance; (2) the driving force of enterprise R&D expenditure on high-quality development is weak, the proportion of R&D expenditure is small and fails to translate into actual benefits, and the innovation ability of enterprise management is insufficient. Based on these findings, three lean paths are designed for the high-quality development of China’s logistics enterprises.

## Figures and Tables

**Table 1:** Evaluation system for high-quality development of logistics enterprises.

	Primary Indicator	Secondary Indicators	Code	Calculation Formula	Source of Literature
Measuring corporate performance indicators	Y1 Social recognition (SR)	Y11 Brand perception	BP	Intangible assets/assets total	[19,20]
		Y12 Social contribution rate	SCR	(taxes paid + employees paid and cash paid for employees + total annual dividends) / main business income	[21,22,23]
		Y13 Corporation value	EV	Market value	[24,25]
	Y2 Scale benefit (SM)	Y21Total assets	TA	Get data directly from financial statements	[21,26,27]
		Y22 Main business cost ratio	OCR	Main business cost/main business income	[24,28,29]
		Y23 Per capita profit	PCP	Net profit/total number of employees	[30]
	Y3 Innovation performance (IP)	Y31 Number of patents	NOP	Get data directly from financial statements	[31,32]
		Y32 Market Development	MD	Sales expenses/total operating income	[32,33]
		Y33 Intangible asset growth rate	IAGR	(Intangible assets of the previous period of intangible assets in the current period)/intangible assets of the previous period	[34]
Affecting corporate performance indicators	X1 Innovation investment (II)	X11 Employee structure	SS	Number of employees with a bachelor degree or above/total number of employees	[24,26,27]
		X12 R&D staff	RDP	Number of R&D staff/total number of employees	[26]
		X13 R&D expenses	RDE	Total R&D expenditure/operating income	[26,35]
	X2 Operational capability (OC)	X21 Fixed asset turnover	FAT	Sales revenue/average net value of fixed assets	[26,36]
		X22 Roe	ROE	After-tax profit/net assets	[27,37,38]
		X23 Assets and liabilities	ALR	Total liabilities/total assets	[33]

**Table 2:** Model regression results.

	Model 1	Model 2	Model 3	Model 4	Model 5
variable	BP	SCR	EV	TA	OCR
SS	−0.026^#^ (−1.588)	0.040 (0.428)	0.123^***^ (4.574)	0.061^***^ (4.440)	0.000 (0.034)
RDP	−0.053 (−1.124)	0.074^*^ (1.839)	−0.177^***^ (−4.639)	−0.033^**^ (−2.292)	0.027^#^ (1.617)
RDE	0.036 (0.553)	0.101^*^ (1.713)	−0.024 (−0.374)	0.007 (0.427)	−0.173^***^ (−8.065)
FAT	−0.007 (−0.279)	0.091^**^ (2.009)	−0.015 (−0.283)	−0.017 (−0.377)	−0.042 (−0.823)
ROE	0.062^***^ (3.527)	−0.021 (−0.672)	0.117^**^ (2.006)	0.065^***^ (3.023)	−0.115^***^ (−4.928)
ALR	−0.009 (−0.257)	−0.485^**^ (−2.568)	−0.068 (−0.498)	0.069 (1.314)	0.102^***^ (3.969)
C	0.152^***^ (8.114)	0.600^***^ (4.845)	0.086 (1.357)	0.036^*^ (1.757)	0.740^***^ (42.749)
Individual effect	fixed	fixed	fixed	fixed	fixed
$R^{2}\_a$	0.937	0.868	0.674	0.974	0.885
	**Model 6**	**Model 7**	**Model 8**	**Model 9**	**Model 10**
variable	PCP	NOP	MD	IAGR	EV
SS	0.083^**^ (2.207)	−0.064^**^ (−2.318)	0.022 (0.639)	0.066 (1.763)	
RDP	0.164^*^ (1.817)	−0.015 (−0.388)	0.111^**^ (2.444)	0.138^**^ (2.193)	
RDE	−0.107^***^ (−2.703)	−0.085 (−1.071)	0.075 (0.815)	−0.170* (−1.727)	
FAT	−0.471 (−1.357)	−0.009 (−0.210)	0.029 (0.409)	−0.449 (−1.416)	
ROE	0.324^***^ (8.150)	−0.020 (−0.464)	−0.144^***^ (−3.661)	0.274^***^ (5.317)	
ALR	−0.228^***^ (−6.925)	0.020 (0.141)	−0.369^***^ (−3.158)	−0.050 (−0.696)	−0.200^**^ (−2.422)
BP					0.444^***^ (4.883)
SCR					−0.174^*^ (−1.849)
TA					1.525^***^ (3.928)
C	0.074^***^ (3.666)	0.109^#^ (1.577)	0.319^***^ (6.500)	−0.039 (−1.180)	0.042 (0.410)
Individual effect	fixed	fixed	fixed	fixed	fixed
$R^{2}\_a$	0.805	0.503	0.816	0.169	0.748

Note: ***, **, * and # respectively indicate significant levels at 1%, 5%, 10% and 15%.

**Table 3:** Model regression results.

	Y1	Y2	Y3
X1	−0.198*** (−3.453)	−0.198^**^ (−2.207)	0.083^*^ (1.770)
X2	−0.144** (−2.093)	−0.067 (−0.624)	0.030 (0.545)
C	0.206*** (13.963)	0.179^***^ (7.710)	0.020^*^ (1.675)

Note: ***, **, * respectively indicate significant levels at 1%, 5%, 10% and 15%.

## References

[1] Ze S. (2015). On the Key Points of Advancing Chinese Logistics Industry in the 13th Five-year Plan Period. Financ. Trade Econ..

[2] Zhang Z.M., Han B. (2018). The Decomposition of “Logistics Cost as a Percent of GDP” and the Comparison between China and America. China Bus. Mark..

[3] Yin Y. (2012). The Analysis on the Problems with China’s Logistics Industry. China Bus. Mark..

[4] Zhao W.Y., Zeng Y.M. (2011). Construction and Design of Evaluation Index System of Innovative Enterprises on Innovative Capacities. Sci. Technol. Manag. Res..

[5] Wang T.N., Li Y.J., Liu J. (2006). Application Research of Enterprise Informationization Performance Evaluation Based on BSC. China Soft Sci..

[6] Ren B.P., Wen F.A. (2018). The Criteria, Determinants and Ways to Achieve High-quality Development in China in the New Era. Reform.

[7] He L.F. (2018). In-depth implementation of the new development concept to promote high-quality development of China’s economy. Macroecon. Manag..

[8] Li W. (2018). Promoting China’s steady march toward high-quality economic development. Wisdom China.

[9] Feng Q.B. (2018). Five characteristics and five approaches of China’s high-quality economic development. Chin. Cadres Trib..

[10] Jia H.Q. (2018). “Four necessities” for achieving higher quality development. Governance.

[11] SU Y.Y., Zhang X.Q., Zhou Y.L. (2015). Performance evaluation of green development strategy of express delivery enterprises based on BSC. Enterp. Econ..

[12] Le X.P. (2016). Transformation and Upgrade of Cold Chain Logistics Enterprises under Reform of the Supply Front. Railw. Transp. Econ..

[13] Shi J.M. (2016). China’s Logistics Enterprises’ Countermeasures of Supply Side Structural Reform. China Bus. Mark..

[14] Xie S.X., Shuai S.Y. (2017). Research on the Strategic Development Path and Tactics Innovation of Logistics Enterprises under the Background of Supply-side Reform in China. China Bus. Mark..

[15] Shi G.Y., Liu G.F., Liang Y.J. (2008). Evaluation of the Market Value Management in Chinese Listed Companies. Chin. J. Manag..

[16] Tether B.S. (2005). Do services innovate differently Insights from European innovation survey. Ind. Innov..

[17] Swamidass P.M. (2003). Modeling the adoption rates of manufacturing technology innovations by small US manufacturers: A longitudinal investigation. Res. Policy.

[18] Liu Q., Zhan H.Y. (2011). Empirical analysis of capital structure influencing factors of real estate listed companies. China Econ..

[19] Ling S.A. (2004). Comprehensive Logistics Strategy of Large Container Shipping Enterprises in China. Master Thesis.

[20] Zhang J., Deng H. (2013). Key Dimensions of Brand Value Co-creation and Its Im-pacts upon Customer Perception and Brand Performance: An Empirical Research in the Context of Industrial Services. Nankai Bus. Rev..

[21] Yi B.N., Han Q.L. (2012). An Empirical Analysis of Private Corporate Social Responsibility and Financial Performance. J. Cent. South Univ. (Soc. Sci.).

[22] Liu C.C., Kong X.T. (2006). Positive research on Society responsibility Accounting Information Highlight-the Empirical Data From 2002 to 2004 on the Shanghai Stock Exchange. Account. Res..

[23] Deng A.M., Yang C.C., Fu Z.M. (2010). The Evaluation of Cool-Chain 3PL Enterprises: An Application of the Extension Method. Theory Pract. Financ. Econ..

[24] Cordero R. (1990). The measurement of innovation performance in the firm: An overview. Res. Policy.

[25] Collier D.W. (1977). Measuring the performance of R&D department. Res. Manag..

[26] China Federation of Logistics and Purchasing Logistics Enterprise Classification and Evaluation Index. https://wenku.baidu.com/view/9490513f4531b90d6c85ec3a87c24028915f85e8.html.20.

[27] Tian H.Y. (2010). Research on the evaluation index system of logistics enterprise operation and management based on SCOR model. Reform Econ. Syst..

[28] Guo R., Zhang Y., Wu X. (2005). The Connotation and Evaluating Method on Sustainable Growth of Firms. Soft Sci..

[29] Ding B., Qv H.M. (2014). Evaluation of operating efficiency of listed logistics enterprises in China and analysis of influencing factors. Mod. Manag..

[30] Xu L.P., Jiang X.R., Yin C. (2015). Research on evaluation index system of enterprise innovation ability. Sci. Res. Manag..

[31] Chen J., Qiu J.M., Shen H.H. (2007). The effect of technical learning on innovative performance. Stud. Sci. Sci..

[32] Hagedoorn J., Cloodt M. (2003). Measuring innovative performance: Is there an advantage in using multiple indicators. Res. Policy.

[33] Pan Y.X., Huang Y.M., Qi E.S. (2006). Chinese manufacturing industry development strategy from the financial perspective. Gray Syst. Theory Appl..

[34] Bao X.Z., Huo H.H., Xu K. (2018). Empirical Study of Intellectual Property Value Index for High-tech Enterprises. J. Intell..

[35] Zhu N.P., Zhu L., Kong Y.S., Shen Y. (2014). Study on Interaction of Technological Innovation Investment and Corporate Social responsibility on Corporate Financial Performance. Account. Res..

[36] Jiang T.S. (2007). Study on the Operation Performance Evaluation System of Highway Transport Enterprise Based on DEA. Master Thesis.

[37] Thor C.G. (1994). The Measures of Success: Creating a High Performance Organization.

[38] Wang D.P., Zai S.Q. (2005). Research on Evaluating Index of TPL Enterprises’ Competitiveness. Theory Pract. Financ. Econ..

[39] Beal R.M., Yasaiardekani M. (2000). Performance Implications of Aligning CEO Functional Experiences with Competitive Strategies. J. Manag..

[40] Jia J.F., Tang G.Y., Li J.P., Wang W.J., Shan X. (2015). The Impact of Manager Competency and Strategic Orientation on Firm Performance. Manag. World..

[41] Wang C.H., Zhang J., Ma J. (2017). Research on the Impact of Lean Inventory Management on Corporate Performance-An Empirical Test from Chinese Manufacturing Listed Companies. Manag. Rev..

[42] Schumpeter J.A. (1986). The Dynamics of Market Economies.

[43] Yang Z. (2018). Environmental protection investment, technological innovation and enterprise market value. Commun. Financ. Account..

[44] Cheng H.W., Zhang Y.H., Chang Y. (2006). An Empirical Study on the Relationship Between R&D Inputs and Performance. Sci. Manag. Res..

[45] Liang L.X., Yan S.D. (2006). Empirical Research on the R&D expenditure and Its Economic Effect of Listed Companies. Sci. Manag. Sci. Technol..

[46] Wang Y., You C. (2009). Empirical research on the correlation between R&D input and performance: Based on panel data of listed companies on the board of small and medium-sized enterprises. Commun. Financ. Account..

[47] Koellinger P. (2008). The relationship between technology, innovation, and firm performance: Empirical evidence from e-business in Europe. Res. Policy.

[48] Chen Y., Zhang B. (2013). Theoretical framework and system of enterprise innovation based on ownership perspective. Econ. Perspect..

[49] Tou Z.B. (2006). Impact of asset structure of listed automobile manufacturing enterprises on enterprise performance. Financ. Account. Mon..

[50] Xu Y.K., Wang Z.T. (2001). Information disclosure of intangible assets and its value correlation-empirical data from Shanghai stock market. Account. Res..

[51] Moskowitz M. (1972). Choosing Socially Responsible Stocks. Bus. Soc. Rev..

[52] Ruf B.M., Muralidhar K., Brown R.M., Janney J.J., Paul K. (2001). An Empirical Investigation of the Relationship between Change in Corporate Social Performance and Financial Performance: A Stakeholder Theory Perspective. J. Bus. Ethics.

[53] Vance S. (1975). Are Socially Responsible Corporations Good Investment Risks. Manag. Rev..

[54] Chen S.Q. (2018). Correlation between R&D investment and corporate financial performance-based on analysis of high-tech enterprises. Res. Dev..

[55] Gao T.M. (2009). Econometric Methods and Modeling.

[56] Huang Y.M., Qi E.S. (2015). Predicament and Way of China’s Manufacturing Industry Based on IE. Sci. Sci. Manag. S. T..

